# The emerging roles of exosomes in the modulation of immune responses in cancer

**DOI:** 10.1186/s13073-018-0535-4

**Published:** 2018-03-26

**Authors:** Paul Kurywchak, Jena Tavormina, Raghu Kalluri

**Affiliations:** 1 Department of Cancer Biology, University of Texas MD Anderson Cancer Center, Houston, TX 77054 USA; 2 The University of Texas MD Anderson Cancer Center, UT Health Graduate School of Biomedical Sciences, Houston, TX 77030 USA

**Keywords:** Exosomes, DNA, Cancer, Innate immunity, STING, Biomarker, Drug delivery

## Abstract

Exosomes are promising tools for improving cancer care, but conversely may also contribute to tumor progression. Here, we highlight recently discovered roles of exosomes in modulating immune responses in cancer, with emphasis on exosomal surface proteins and on RNA and DNA content. We also discuss how exosomes could be exploited as biomarkers and delivery vehicles in cancer therapy.

## Exosomal surface proteins and RNA can regulate immune responses in cancer

Exosomes are a subset of extracellular vesicles with endosomal origin that collectively reflect the contents of their parental cells. A growing body of research has investigated the physiological functions of specific exosomal components, such as proteins, RNA, and DNA. This work has revealed sophisticated mechanisms involving the suppression of tumor development by immune cell-derived exosomes, and the contribution of cancer cell-derived exosomes to tumor development. Originally, exosomes from B lymphocytes were found to use major histocompatibility complex (MHC) class I and II proteins on their surface to perform antigen presentation—work that has been expanded on by others to demonstrate the antitumor effect of immune cell exosomes (recently reviewed in [1]). Recent research has also demonstrated how tumor cells can produce or promote the production of immune-modulating exosomes, and how the contents of these exosomes support cancer progression [2, 3].

Hoshino et al. [2] demonstrated that exosomes may be potent facilitators of metastatic colonization in secondary organs. They concluded that exosomal integrins dictate the cellular uptake of exosomes, which leads to Src activation and upregulation of S100 genes in resident cells at tropic sites of metastasis. These pro-migratory and inflammatory signals then have extracellular immune effects, such as recruiting bone marrow-derived myeloid cells that further stimulate inflammation [2]. Similarly, Nabet et al. [3] discovered that breast cancer cells can stimulate NOTCH-MYC signaling in activated fibroblasts within the tumor microenvironment, producing exosomes containing unshielded (activated) *RN7SL1*, an endogenous RNA that is normally shielded (deactivated) by RNA-binding proteins. This unshielded RNA functions as a damage-associated molecular pattern (DAMP) and drives inflammation signaling in splenic myeloid cells. In addition, exosomes carrying unshielded *RN7SL1* promoted tumor progression and metastasis through dependent activation of the pattern recognition receptor (PRR) retinoic acid-inducible gene I (RIG-I) [3]. In these cases, cancer cells co-opt surrounding and distant microenvironments to promote cancer progression through stimulation of antiviral immune pathways. Conversely, in a lung cancer model, Gao et al. [4] showed how tumor-derived exosomes can have a suppressive effect on innate immunity that reduces protection against viral infection. This immunosuppressive effect was mediated by the delivery of exosomes carrying activated epidermal growth factor receptor (EGFR) to host macrophages, which resulted in the repression of interferon regulatory transcription factor 3 (IRF3) and type 1 interferon (IFN) expression [4]. Unlike the previously mentioned studies that describe how tumor cell-derived exosomes promote inflammation, this work shows how exosomes can have different functions depending on the cell type with which they interact.

**Fig. 1:**
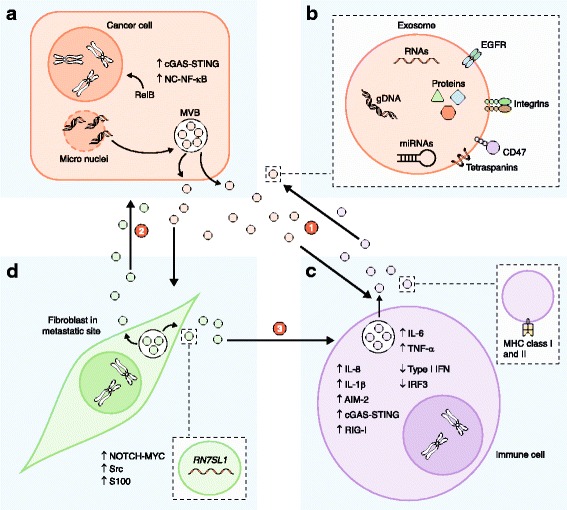
Exosomes from cancer cells modulate immune responses and can co-opt local and distant normal cells to further promote tumor progression. (1) Cancer cells (**a**) release exosomes (**b**) that carry damage-associated molecular patterns (DAMPs) such as DNA and RNA to myeloid cells (**c**) which activate the intracellular virus-sensing pathways cyclic GMP-AMP synthase—stimulator of interferon genes (cGAS-STING), retinoic acid-inducible gene I (RIG-I), and Absent in melanoma 2 (AIM-2), and stimulate the production of inflammatory cytokines such as interleukin (IL)-6, tumor necrosis factor (TNF)-α, IL-8, and IL-1β [6, 7]. Conversely, activated epidermal growth factor receptor (EGFR) on the surface of breast cancer exosomes (**b**) can suppress antiviral innate immunity in dendritic cells (**c**) through the kinase MEKK2, which prevents activation of interferon regulatory transcription factor 3 (IRF3) and type 1 interferon (IFN) expression [4]. Immune cells (**c**) such as B lymphocytes produce major histocompatibility complex (MHC) class I- and II-containing exosomes that can have antitumor effects through activation of CD8^+^ T cells, for example [1]. (2) Cancer cells (**a**) can also indirectly alter immune cells (**c**) through the integrin-based interaction of exosomes (**b**) with surrounding and distant normal fibroblasts (**d**) and epithelial cells [2, 3]. (3) Subsequently, these interactions can promote tumor growth and metastasis by driving inflammation of myeloid cells (**c**) through antiviral immune mechanisms, involving activation of the pattern recognition receptor (PRR) RIG-I in the cancer cells (**a**) [3]. gDNA, genomic DNA; MVB, multivesicular body

The disparities between these findings further highlight the need for greater understanding of exosome-mediated effects; for example, there is a need to determine whether these effects are context- or model-specific or whether they have similar functions in humans. Nevertheless, these studies and others related to exosome-mediated immune modulation highlight the importance of DAMPs and the PRRs that recognize them. This showcases how tumor cells can mimic viral mechanisms to modulate immune responses and to promote their survival and expansion systemically (Fig. 1).

## Exosomal DNA may also contribute to the modulation of tumor immunity

In addition to RNA, exosomes have been found to contain genomic DNA, which collectively spans the entire genome and reflects the genetic status of the parental cell [5]. Work from numerous groups has also shown that exosomal DNA levels are lower in non-transformed cell lines and in circulating exosomes from healthy individuals compared with exosomes from cancer cell lines and cancer patients [5]. Little is known about the function of exosomal DNA in comparison to exosomal RNA, but recent research has provided insight into the effects of exosomal DNA on immune cell responses.

Recent work by Takahashi et al. [6] showed that human fibroblast exosomes remove harmful cytosolic DNA to maintain cellular homeostasis, and that when exosome production was inhibited genomic DNA accumulated in the cytoplasm. This led to a reactive oxygen species (ROS)-dependent DNA damage response (DDR) that was mediated by the cytosolic DNA-sensing pathway cGAS-STING (cyclic GMP-AMP synthase—stimulator of interferon genes), resulting in cell cycle arrest or apoptosis [6]. Kitai et al. [7] demonstrated that the treatment of breast cancer cells with the topoisomerase I inhibitor topotecan (an antitumor chemotherapeutic that triggers DNA double-strand breaks and DDR) significantly increased exosomal DNA production and led to dendritic cell activation through cGAS-STING signaling, showing that exosomal DNA can also activate innate antiviral immune cell responses. Similarly, Lian et al. [8] showed that exosomal DNA mediates intestinal inflammation that can lead to severe diarrhea in patients treated with the chemotherapeutic irinotecan, causing AIM-2 (Absent in melanoma 2) inflammasome pathway activation in dendritic cells and the production of the cytokines IL-1β and IL-18. These studies directly implicate exosomal DNA in altering innate immune responses and suggest that its production is in response to cellular stresses such as DDR activation.

Interestingly, Bakhoum et al. [9] recently reported that chromosomal instability in cancer cells and errors in chromosomal segregation lead to cytosolic DNA accumulation and subsequent cGAS-STING activation, promoting tumor cell invasion and metastasis. To date, a link between chromosomal instability and exosomal DNA accumulation has not been directly established, but the research highlighted here suggests that such a link probably exists. Taken together, these studies implicate exosomes as modulators of innate immunity through activation of the c-GAS-STING or AIM-2 pathways (Fig. 1). Precise mechanisms that dictate DNA packaging into exosomes remain to be discovered, but additional research is likely to identify the key regulators that are involved.

## Translational opportunities for exosomes and exosomal DNA

Exosomes may be important facilitators of intercellular communication, but it is probable that they can also be exploited as treasure troves of diagnostic and prognostic biomarkers that can be collected serially and non-invasively. The identification of unique exosomal patterns or features in pathological states could be used to develop reliable indicators of disease status. In the case of exosomal DNA, an average fragment length that is greater than that of circulating free DNA provides advantages for detecting tumor-specific mutations and rearrangements [5]. One example of the clinical potential of exosomal DNA was reported by our laboratory [10], which revealed that oncogenic *KRAS*^G12D^ and *TP53*^R273H^ mutations could be detected in serum exosomes from patients with pancreatic cancer using digital PCR [10]. In addition, exosomal DNA is being actively used for next-generation sequencing and, owing to the extensive heterogeneity often observed in solid tumors, exosomal DNA may more accurately represent overall tumor genetics than do the small tissue biopsies that are often used for genetic evaluation.

Lastly, an exciting application for exosomes that has already begun to be explored in clinical trials involves their use as vehicles for carrying therapeutic payloads. Another recent study from our laboratory showed that oncogenic *KRAS* can be targeted directly and specifically in pancreatic cancer cells using exosomes loaded with short interfering RNA, which had previously been very difficult to achieve [11]. Experiments from this study revealed that ablating oncogenic *KRAS* as a single target significantly improved overall survival in multiple animal models. This strategy will need to be fully evaluated for efficacy in clinical trials, but could provide significant improvement over current therapies for pancreatic cancer, and may be adaptable to improve therapeutic outcomes in other cancers. In addition, areas of active investigation are the use of exosomes to carry different payloads, such as chemotherapeutics or even CRISPR-Cas9 for genome editing, and to enhance immunotherapy.

The primary advantage of using exosomes for drug delivery is that exosomes are immunogenic, partly because they express CD47, an integrin-associated transmembrane protein that prevents phagocytosis by monocytes [11]. In addition, unlike synthetic drug delivery vehicles, exosomes contain other membrane proteins, such as integrins, that may enhance endocytosis and delivery of their payload to recipient cells. The primary limitation that will need to be overcome is determining the underlying mechanisms that govern exosome targeting, and that therefore inform the required payload potency for each proposed therapeutic indication. In the study by Kamerkar et al. [11], enhanced exosome targeting to pancreatic cancer cells was shown to be mediated by *KRAS*-driven micropinocytosis in cancer cells. Manufacturing and regulatory guidelines are still being developed, but efforts are already being made by both biotechnology companies and academic institutions to conduct exosome-based clinical trials.

## Concluding remarks and future prospects

Recent research has provided insights into the different ways in which exosomal contents can modulate immune cell function to influence cancer progression, and how exosomes may also have translational importance. To date, there are no specific inhibitors of exosome production, or models for differentiating and tracking different populations of extracellular vesicles. Models are actively being developed to achieve this and will greatly improve our ability to characterize the behavioral dynamics and importance of these vesicles in different biological contexts. In addition, accumulating preclinical research has revealed many clinical applications for exosomes, both for cancer detection and for cancer therapy. Although the field is relatively young, efforts are underway to validate these biomarkers in large patient cohorts and establish Good Manufacturing Practice (GMP) conditions for the development of clinical-grade exosomes. There remain many unsolved questions and hurdles to overcome, but research over the next several years will provide further insight into the importance of exosomes as biological and theranostic factors.

## Abbreviations

AIM-2: Absent in melanoma 2
cGAS-STING: Cyclic GMP-AMP synthase—stimulator of interferon genes
DAMP: Damage-associated molecular pattern
DDR: DNA damage response
IRF3: Interferon regulatory transcription factor 3
MHC: Major histocompatibility complex
PRR: Pattern recognition receptor
